# The Role of Imaging in Ventricular Tachycardia Ablation

**DOI:** 10.3390/diagnostics15151973

**Published:** 2025-08-06

**Authors:** Pasquale Notarstefano, Michele Ciabatti, Carmine Marallo, Mirco Lazzeri, Aureliano Fraticelli, Valentina Tavanti, Giulio Zucchelli, Angelica La Camera, Leonardo Bolognese

**Affiliations:** 1 Cardiovascular Department, San Donato Hospital, 52100 Arezzo, Italy; michele.ciabatti@uslsudest.toscana.it (M.C.); carmine.marallo@gmail.com (C.M.); mircolazzeri@gmail.com (M.L.); aureliano.fraticelli@uslsudest.toscana.it (A.F.); leonardobolognese@hotmail.com (L.B.); 2 Radiology Department, San Donato Hospital, 52100 Arezzo, Italy; valentina.tavanti@uslsudest.toscana.it; 3 Second Division of Cardiology, Cardio-Thoracic and Vascular Department, Pisa University Hospital, 56124 Pisa, Italy; g.zucchelli@ao-pisa.toscana.it; 4 Biosense Webster, Johnson & Johnson Medial SpA, 00071 Pomezia, Italy; alacamer@its.jnj.com

**Keywords:** Imaging, catheter ablation, ventricular tachycardia, cardiac magnetic resonance, computed tomography, intracardiac echocardiography

## Abstract

Ventricular tachycardia (VT) remains a major cause of morbidity and mortality in patients with structural heart disease. While catheter ablation has become a cornerstone in VT management, recurrence rates remain substantial due to limitations in electroanatomic mapping (EAM), particularly in cases of deep or heterogeneous arrhythmogenic substrates. Cardiac imaging, especially when multimodal and integrated with mapping systems, has emerged as a critical adjunct to enhance procedural efficacy, safety, and individualized strategy. This comprehensive review explores the evolving role of various imaging modalities, including echocardiography, cardiac magnetic resonance (CMR), computed tomography (CT), positron emission tomography (PET), and intracardiac echocardiography (ICE), in the preprocedural and intraprocedural phases of VT ablation. We highlight their respective strengths in substrate identification, anatomical delineation, and real-time guidance. While limitations persist, including costs, availability, artifacts in device carriers, and lack of standardization, future advances are likely to redefine procedural workflows.

## 1. Introduction

Ventricular tachycardia (VT) remains a significant cause of cardiovascular morbidity and mortality, particularly in patients with structural heart disease [1].

Catheter ablation has emerged as a milestone treatment modality for this potentially life-threatening arrhythmia due to increasing evidences of its superiority over antiarrhythmic drugs [2,3,4,5,6]. However, despite technological advancements and increasing operator experience, the recurrence rate following ablation procedures remains significant [3].

Electroanatomic mapping (EAM), the gold standard for arrhythmogenic substrate characterization, has some limitations. Activation mapping is effective only if the arrhythmia is inducible, sustained, and well-tolerated. The evaluation of low-voltage areas depends on factors such as catheter tip orientation, tissue contact, and far-field effects from healthy myocardium. Additionally, substrate mapping and pace mapping are less reliable when the arrhythmogenic substrate is located intramyocardially [7].

In recent years, cardiac imaging has assumed an increasingly relevant role in enhancing both the efficacy and safety of VT ablation procedures.

The expert consensus statement on VT ablation recommends integrating preprocedural imaging for accurate identification of potential arrhythmogenic substrates and procedural planning [3].

Multimodal imaging techniques, including transthoracic and intracardiac echocardiography (ICE), cardiac magnetic resonance imaging (CMR), computed tomography (CT), and positron emission tomography (PET), have shown to be invaluable tools in the periprocedural evaluation of patients undergoing VT ablation. These advanced imaging-based approaches provide

1. Detailed characterization of the arrhythmogenic substrate;
2. Optimal planning of access strategies;
3. Real-time guidance during the procedure;
4. Assessment of ablation efficacy.

The integration of imaging-derived information with EAM systems has led to a more precise and tailored approach to VT ablation. This synergy has significantly increased our understanding of the complex substrates underlying VT and has enabled more targeted and effective ablation strategies.

This review aims to critically examine the current role and future perspectives of various imaging modalities in facilitating and optimizing VT ablation procedures. We will explore the strengths and limitations of each imaging technique, discuss their integration into clinical practice, and highlight emerging technologies that promise to further refine our approach to VT ablation.

## 2. Pre-Procedural Imaging Assessment

**Figure 1:**
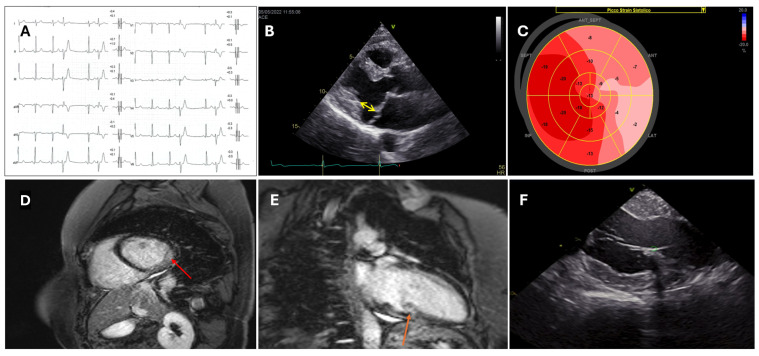
A 35-year-old female with mitral valve prolapse presented an elevated ventricular arrhythmic burden and underwent cardiological evaluation. (**A**) A resting ECG showing the presence of PVCs with right bundle branch block-like morphology (qR in lead V1) and superior axis; (**B**) TEE showed the presence of bileaflet mitral valve prolapse with mitral annular disjunction (yellow arrow); (**C**) reduced strain values in the lateral wall shown in speckle tracking echocardiography; (**D**) and (**E**): CMR demonstrated significant midwall LGE in inferolateral segments (red arrow) and in the postero-medial papillary muscle (orange arrow); (**F**) after ineffective anti arrhythmic treatments, the patient underwent PVC ablation at the posterior–medial papillary muscle. Adequate contact between the ablation catheter and the papillary muscle was assured through ICE. PVC, premature ventricular complex; TEE, transesophageal echocardiography; CMR, cardiac magnetic resonance; LGE, late gadolinium enhancement; ICE, intracardiac echocardiography.

Transthoracic echocardiography (TTE) represents an unexpensive and widely available imaging tool. It can provide a wide range of information regarding biventricular function, dimensions, and valvular physiology. Reduced left ventricular ejection fraction (LVEF) and functional right ventricular indexes have been associated with extensive substrates and worse prognosis in patients with ischemic heart disease or cardiomyopathies undergoing VT ablation [8,9]. In cases of VT originating from the valvular apparatus, preprocedural echocardiography could provide important clues regarding the anatomical structures involved (Figure 1B,C). The presence of regional left or right ventricular wall motion abnormalities (i.e., segmental a-dyskinesia, regional wall thinning) could suggest the presence of underlying pathological substrate. However, detection of regional dysfunction is affected by significant inter-operator variability and low reproducibility. Over the years, speckle tracking techniques have demonstrated higher sensitivity in detecting subtle cardiac wall motion abnormalities compared to standard echocardiography [10]. Impaired echocardiographic endocardial and epicardial strain values have been associated with pathological bipolar and unipolar EAM areas in patients with ischemic heart disease undergoing ventricular arrhythmia ablation [11]. Deformation imaging analysis is able to reveal subtle pathological substrate in patients with cardiomyopathies, and impaired strain values have been associated with worse outcomes in subjects affected by arrhythmogenic cardiomyopathy (AC) or sarcoidosis [12,13,14].

**Figure 2:**
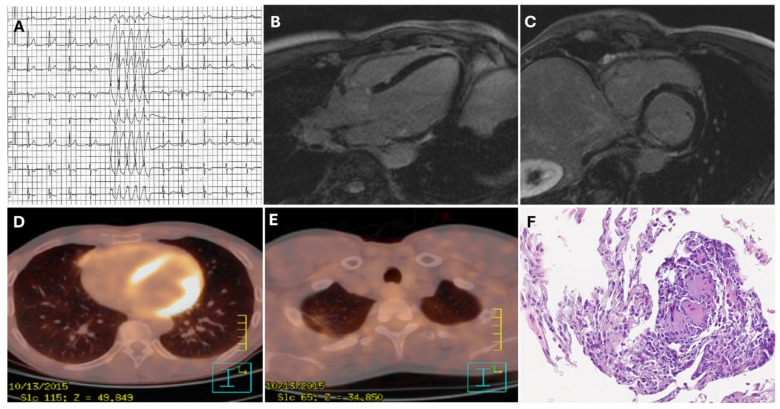
A 55-year-old male with non-ischemic cardiomyopathy was evaluated for catheter ablation of symptomatic PVCs and NSVT (**A**); (**B**,**C**) CMR demonstrated the presence of patchy LGE in the interventricular septum and inferolateral wall; (**D**,**E**) intense 18-FDG uptake was detected by a PET scan in the interventricular septum and inferolateral wall and in the apical right pulmonary lobe; (**F**) inflammatory infiltrates and non-caseating granulomas were identified through histology after pulmonary lymphonode biopsy, compatible with pulmonary sarcoidosis. After steroid therapy, the patient experienced significant symptomatic improvement and reduction in arrhythmic burden; therefore, catheter ablation was postponed. PVCs, premature ventricular complexes; NSVT, non-sustained ventricular tachycardia; CMR, cardiac magnetic resonance; LGE, late gadolinium enhancement; 18-FDG PET, 18-fluorodeoxyglucose positron emission tomography.

Cardiac magnetic resonance (CMR) can provide comprehensive biventricular functional assessment together with multiparameter tissue characterization, a prominent factor in patients with arrhythmic phenotypes [1]. Left and right ventricular ejection fraction are established predictors of short- and long-term adverse outcomes after VT ablation [3,15,16,17]. T2-weighted sequences and T2 mapping techniques can detect edema in patients with inflammatory cardiomyopathies, thereby guiding dedicated diagnostic and therapeutic pathways. Late gadolinium enhancement (LGE) could be an expression of fibrosis, inflammation, or expanded extracellular volume (Figure 1D,E). LGE localization and pattern can provide important clues in the differential diagnosis between ischemic, genetic, and acquired cardiomyopathies (Figure 2B,C) [1,9], and it is crucial in setting up the procedural workflow. Presence and extent of LGE have been extensively associated with adverse arrhythmic events in patients with coronary disease and cardiomyopathies. Moreover, LGE location can suggest a preferential access (endocardial, epicardial, or combined) depending on the predominant underlying substrate [18]. Over the years, high-resolution late-gadolinium-enhanced CMR (LGE-CMR) has emerged as a novel non-invasive tool for detecting conducting channels (CCs) before procedures through pixel signal intensity (PSI) analysis [19,20,21].

Although the clinical benefits of CMR have been widely investigated, it is only in recent years that evidence has emerged demonstrating its safe use and feasibility in the majority of patients with implantable cardioverter defibrillators (ICDs) [22,23]. Beyond safety concerns, a significant obstacle to the utilization of CMR-provided information in device carriers has long been represented by metal-induced hyperintensity artifacts [24,25] on LGE images, which can appear similar to the hyperenhancement of scar tissue. These artifacts especially occur at regions of the heart that are close to the device generator, such as the anterior and lateral walls, and the outflow tracts [26]. Device-related artifacts also appear on cine and perfusion CMR images, even if these are usually less important than those on LGE sequences. Luckily, over the last few years novel LGE-CMR wideband (WB) sequences have been developed to attenuate these artifacts [27,28,29,30,31]. Roca-Luque et al. demonstrated that WB analysis can effectively detect arrhythmogenic CCs even in patients with ICD undergoing VT ablation [28]. Patel et al. [32] recently evaluated a large population of ICD patients undergoing CMR with a WB technique, revealing that 36% had a new or changed diagnosis and 28% experienced management changes, especially those with VT. Patients with LGE had worse outcomes, with a higher incidence of major adverse cardiac events (MACEs). Overall, LGE CMR proved to be highly valuable for clinical decision-making and prognosis in ICD patients.

Tissue characterization of the right ventricle (RV) by CMR is hampered by the reduced thickness of the free wall, and LGE identification is often difficult. CMR-derived feature tracking analysis can detect mild RV abnormalities and has been associated with pathological epicardial and endocardial EAM voltages in patients with arrhythmogenic cardiomyopathy, even in the absence of overt LGE [33]. Catheter ablations of ventricular arrhythmias have been increasingly performed in patients with severe left ventricular (LV) or RV dysfunction, often necessitating intensive care unit support and temporary LV or RV mechanical support. Therefore, novel scores, such as PAINES2D (Pulmonary disease, Age, Ischemic cardiomyopathy, NYHA class, Ejection fraction, Storm, Scar volume, Diabetes), have been developed to predict the risk of hemodynamic decompensation after VT ablation [34]. In this setting, CMR can provide important prognostic information such LVEF, right ventricular function, and scar extent for periprocedural planning in high-risk subjects undergoing complex procedures.

Contrast-enhanced computed tomography (CT) has increasingly been used in many cardiovascular disorders, including coronary artery, valvular, and pericardial diseases [35,36]. Multidetector CT (MDCT) can provide accurate information regarding wall thickness, myocardial perfusion, and fat deposition [35,37]. MDCT can identify scar tissue and border zones which are commonly targeted during VT ablation procedures [38,39,40]. Lipomatous metaplasia can be detected in patients with coronary artery disease and cardiomyopathies [41,42,43,44], and it may play a significant role in promoting scar-related reentry by affecting current leak and conduction velocity [45,46]. Moreover, MDCT can reveal other important prognostic elements, such as coronary artery plaques and unrecognized intracardiac thrombosis. Careful assessment of coronary anatomy and periprocedural multimodality imaging are crucial in the case of an epicardial approach to reduce the risk of complications. Notably, MDCT can represent an appealing option for subjects with ICD affected by VT, even in cases when CMR is contraindicated or impaired by artifacts.

Positron emission tomography (PET) can provide precious insights about metabolic cardiac pathways, especially in the presence of cardiac inflammation by use of 18F-fluorodeoxyglucose (FDG) or other tracers [18,47]. An appropriate low-carbohydrate diet with elevated saturated fat consumption before the exam is fundamental in order to induce a shift in cardiac tissue metabolism and avoid artifacts.

Inflammatory processes detected in patients undergoing PET before ablation led to identification of specific etiologies (such as lymphocytic myocarditis and sarcoidosis) and improved the therapeutic management (Figure 2D,E). Specifically, 18-FDG PET has emerged as a pivotal imaging tool for identifying cardiac and extracardiac sarcoidosis, and it can help in planning optimal diagnostic and therapeutic pathways in these patients [48]. In this setting, RV uptake has been associated with worse prognosis in subjects with cardiac sarcoidosis [49]. It is important to note that FDG-PET can reveal the presence of unrecognized cardiac inflammation, even in patients with arrhythmogenic cardiomyopathies carrying pathogenetic or likely pathogenetic variants [50]. Therefore, identification of inflammatory processes could potentially lead to the use of immunomodulatory therapies in patients with high arrhythmic burden and optimize the timing of catheter ablation (Figure 2). Even if less accurate than other imaging modalities, 18-FDG PET can identify metabolic heterogeneous zones. The burden of the heterogeneous zone has been associated with poor prognosis in patients undergoing VT ablation [51]. However, the resolution of 18-FDG PET is limited compared to MDCT, and it cannot reach the extensive myocardial tissue characterization provided by CMR techniques.

LV thrombosis could represent a significant challenge due to the risk of systemic embolism during ablation procedures, especially in patients with LV aneurysms. TTE presents limitations for detecting small LV thrombi, but contrast-enhanced techniques can significantly improve sensitivity [52]. CMR represents the gold standard for LV or RV thrombi detection due to its excellent discrimination between cardiac muscle, blood pools, and thrombotic material [53,54]. MDCT could represent another promising tool for this aim due to its elevated resolution, especially in patients with intracardiac devices. Recently, dual-energy CT has emerged as a promising strategy for identifying occult intracardiac thrombosis [55]. PET with specific radiotracers targeting active thrombus formation (18F-GP1, which binds to activated platelet glycoprotein IIb/IIIa receptors) represents a novel technique to detect early thrombosis and is currently under evaluation [54].

## 3. Intraprocedural Imaging: Focus on Intracardiac Echocardiography

Intracardiac echocardiography (ICE) has emerged as an invaluable tool in the field of interventional electrophysiology, particularly in the context of VT ablation [52]. This advanced imaging modality offers real-time, high-resolution visualization of cardiac structures, enabling precise catheter navigation and improved procedural outcomes by simply positioning the ultrasound probe in the right atrium and ventricle [53]. Unlike transesophageal echocardiography, ICE can be performed by the operator, without the need for general anesthesia and esophageal intubation [54]. The commonly used technology is phased-array ICE, which consists of a 64-element transducer mounted at the distal end of an 8- or 10-French steerable catheter that can be deflected in four directions (anterior, posterior, right, and left). A wedge-shaped image is generated and displayed on a standard ultrasound workstation. Compared to mechanical rotational systems, phased-array ICE offers several advantages, including greater depth of penetration (up to 15 cm), enhanced maneuverability, and the capability to acquire Doppler and color flow imaging [54,55].

**Figure 3:**
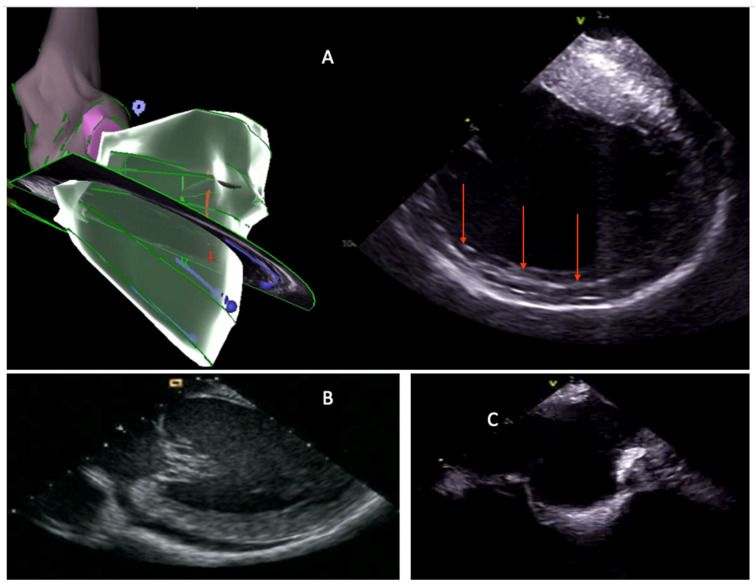
Examples of applications of ICE in VT ablation: (**A**) 3D reconstruction of the left ventricle obtained with the ICE probe positioned in the right ventricle, showing a subendocardial scar area on the lateral wall (red arrows); (**B**) intraprocedural detection of pericardial effusion; (**C**) identification of a large aortic plaque at the sino-tubular junction, which prompted the operator to avoid the transaortic approach and proceed exclusively via the transseptal route. ICE, intracardiac echocardiography; VT, ventricular tachycardia.

ICE-derived anatomical data can be merged with EAM Systems. In the CARTOSOUND module (CARTO, Biosense Webster, Inc., Diamond Bar, CA, USA), a specially adapted ICE catheter (Soundstar) with an embedded positional sensor is employed. The device acquires several planar images at different angles within the cardiac chamber, and the inner wall is outlined either manually or through automated edge-detection algorithms (Figure 3A). These contours are then assembled by the software to form a dynamic three-dimensional model, onto which electrical mapping data is subsequently superimposed. Intracavitary structures can be acquired as separate objects. A green marker is used to display the real-time location of the catheter tip when it intersects the ultrasound beam [54]. This integration allows for a more comprehensive understanding of the cardiac chambers’ anatomical features and enhances the precision of catheter navigation during ablation procedures (Figure 3).

The utility of ICE in VT ablation spans multiple domains, including anatomical delineation, substrate characterization, catheter–tissue contact assessment, and complication monitoring (Figure 1F and Figure 3).

### 3.1. Anatomical Delineation and Catheter Navigation

ICE provides live visualization of cardiac chambers, valvular structures, and intracardiac masses, facilitating accurate catheter positioning and navigation; in VT ablation, it creates detailed imaging of the ventricular anatomy, including critical structures. By delineating the coronary cusps and identifying the origin of the coronary arteries, ICE may eliminate the need for coronary angiography in cases of arrhythmias originating from the coronary cusps. The term “fourth dimension” has been proposed for intracavitary structures such as papillary muscles, false tendons, and the moderator band [56], which can serve as arrhythmogenic foci and are difficult to target during ablation due to their complex 3D geometry, not easily understandable with EAM alone. ICE allows accurate real-time visualization of the anatomical landmarks of these structures, enabling operators to navigate complex anatomies with greater confidence and precision (Figure 1F) [57,58,59,60]. EHRA Expert consensus on catheter ablation of ventricular arrhythmias recommends ICE (Class I, LOE B-NR) to identify and target the papillary muscles and to assess for catheter stability [3].

### 3.2. Substrate Characterization

One of the most significant advantages provided by ICE in VT ablation is its ability to visualize in real time the potential arrhythmogenic substrate. Even without offering the substrate characterization capabilities of CMR and CT, ICE can identify areas of myocardial scarring (Figure 3A) that serve as the substrate for reentrant VT circuits [61,62,63]. These regions typically appear with increased echogenicity, wall thinning, or akinesis. Furthermore, ICE can detect intramural and epicardial substrates, guiding the procedural approach; this capability is particularly valuable in cases of non-ischemic cardiomyopathy [64,65].

### 3.3. Catheter–Tissue Contact Assessment

Optimal catheter–tissue contact is of foremost importance for effective energy delivery and lesion formation during VT ablation. ICE provides real-time visualization of the catheter tip in relation to the myocardium, allowing operators to ensure adequate contact before and during radiofrequency energy application. This feature is especially useful when ablating in areas with complex anatomy or in regions where catheter stability may be challenging, such as papillary muscles. ICE is of critical importance during ablation in the right and left ventricular outflow tracts, as it allows for direct visualization of the valvular apparatus and enables accurate tagging of their position within the EAM system [54,66,67,68].

### 3.4. Complication Preventing and Monitoring

ICE plays a vital role in enhancing the safety profile of VT ablation procedures by enabling real-time monitoring for potential complications. It allows for early detection of pericardial effusion, a harbinger of cardiac perforation (Figure 3B). Additionally, ICE can visualize microbubble formation during radiofrequency energy delivery, which may precede steam pops and tissue overheating [69]. The ability to detect these complications in their nascent stages allows for prompt intervention, potentially mitigating more severe sequelae. Furthermore, the ability to visualize cardiac chambers, access routes, and anatomical alterations in real-time before positioning the ablation catheter can guide the operator in avoiding manipulations of the ablation catheter in areas at risk of complications [70,71]. Figure 3C shows a case of ischemic VT ablation in which the visualization of a large aortic plaque led the operator to avoid the retrograde transaortic approach and to perform the procedure using only the transseptal approach instead.

### 3.5. Radiation Exposure Reduction

The use of ICE in VT ablation procedures has been associated with a significant reduction in fluoroscopy time and radiation exposure for both patients and operators. In some cases, ICE guidance has enabled the performance of “zero-fluoroscopy” VT ablation procedures, which is particularly beneficial for young patients and pregnant women. [66,67,68,72]

### 3.6. Procedural Outcomes and Clinical Impact

Despite the above-mentioned advantages offered by ICE, randomized studies on clinical impact are still lacking. A large retrospective analysis showed that the use of ICE during VT ablation was associated with a lower likelihood of 12-month VT-related readmissions and repeat ablations compared to procedures performed without ICE [73]. In a nationwide Japanese database, the ICE group showed a lower prevalence of cardiac tamponade than the non-ICE group, with no additional clinical advantages [74].

## 4. Intraprocedural Imaging: Focus on CT and CMR

The integration of advanced imaging modalities, particularly MDCT and CMR, into EAM systems represents one of the major technological advancements in the field of transcatheter ablation in recent years. The integration of MDCT and CMR provides helpful information for procedural planning, substrate characterization, and real-time guidance during ablation.

LGE-CMR has emerged as a powerful tool for identifying and characterizing myocardial scar, which serves as the substrate for reentrant VT circuits. LGE-CMR can delineate not only the core scar but also the heterogeneous border zone tissue, which has been shown to correlate well with areas of slow conduction and VT isthmuses identified on EAM.

Compared with CMR, MDCT offers superior spatial resolution, allowing detailed assessment of the myocardial structure [53]. As a consequence, MDCT provides detailed anatomical information, including the coronary artery and venous anatomy, valve apparatus, and the left phrenic nerve, which is particularly valuable in cases of epicardial approach, for enhancing procedural safety [75]. Furthermore, CT can identify areas of epicardial fat, which may affect local electrogram characteristics and ablation efficacy. Even if MDCT provides a high spatial resolution, it exhibits a lower contrast-to-noise ratio within myocardial tissue that can result in less accurate scar characterization compared to CMR [18], which is essential during VT ablation. In the past decade, various studies investigated the utility of importing anatomical and substrate information obtained from CT and CMR into the EAM system. The integration software initially available provided the integration of anatomic features of cardiac chambers, but the segmentation of coronary arteries, the left phrenic nerve and, more importantly, the characterization of myocardial scar were not supported.

After promising evidence was gathered in small preliminary studies [76,77], a growing interest in imaging integration emerged, thanks also to the development of dedicated software enabling the integration of important patient-specific data on cardiac anatomy and structural substrate.

In the study by Komatsu et al. [40], the integration of MDCT wall thickness (WT) with 3D electroanatomic maps was useful to focus mapping and ablation on the culprit regions in postinfarction VT. A significant correlation was found between the areas of WT < 5 mm and low endocardial voltage, but no such correlation was found in the epicardium. The vast majority (87%) of areas of low voltage and local abnormal ventricular activity (LAVA) were located in areas with WT < 5 mm or at their borders, and very late LAVAs (>100 msec after QRS complex) were almost exclusively detected within the thinnest area (<3 mm), showing a correlation between regional myocardial WT, low-voltage regions, and the distribution of LAVA critical for the generation and maintenance of postinfarction VT. However, despite a good correlation, areas with WT < 5 mm were consistently smaller than the endocardial low-voltage area.

Yamashita et al. [78] first reported the systematic use of imaging integration with CT or CMR in a substantial number of patients (116) undergoing the catheter ablation of VT scars related to various etiologies. Image processing was obtained with a dedicated software, and all segmented structures were exported in the form of 3D meshes and loaded into 3D mapping systems.

Imaging integration allowed the identification of 89% of critical isthmuses and 85% of local abnormal ventricular activities (LAVAs). CMR proved to be superior to CT in detecting arrhythmic substrates, but its use was limited to 30 patients due to exclusion criteria like the presence of ICDs. CT, on the other hand, offered higher spatial resolution, enabling the detailed visualization of cardiac structures, including the phrenic nerve and coronary arteries, which influenced epicardial ablation strategies in a significant number of cases. Imaging also prompted additional mapping in over half of the patients and epicardial access in a third, with CMR producing significantly fewer false positives compared to CT. The key messages of this study are that CMR is superior to CT in detecting arrhythmic substrates, although its feasibility is limited to patients without implanted devices. CT, on the other hand, excels in providing detailed anatomical resolution, allowing for the precise identification of cardiac structures, but it is less accurate in identifying arrhythmic substrates.

Despite these limitations, MDCT remains a valuable alternative to CMR, provides important information for the identification and characterization of an arrhythmogenic substrate in postinfarction VT, and can help focus mapping and ablation on the culprit regions [79].

**Figure 4:**
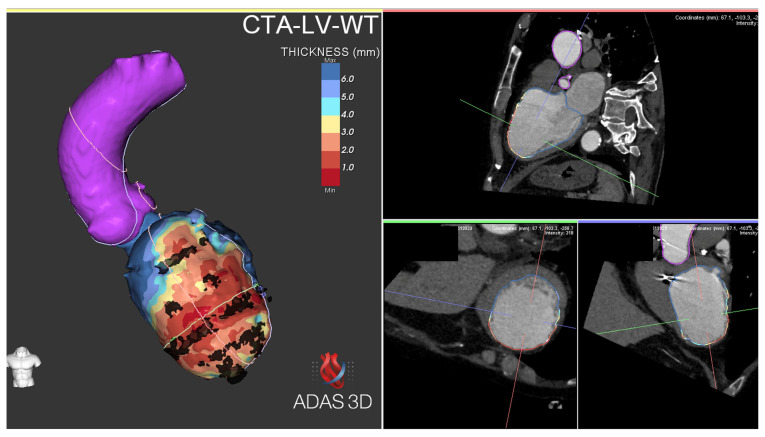
MDCT imagery from an ischemic patient post-processed with a dedicated software (ADAS 3D). A Three-dimensional reconstruction of the aorta (pink) and left ventricle with lipomatous metaplasia (transparent black) and wall thickness analysis. LV wall thickness is shown as a color map (from blue > 6 mm to red < 1 mm). LV, left ventricle; MDCT, multidetector CT.

Lipomatous metaplasia plays a role in the arrhythmogenic substrate of both ischemic and non-ischemic cardiomyopathies. This pathological process, characterized by the replacement of myocytes with adipose tissue within fibrotic scars [41,42,43,44], alters the electrophysiological properties of the myocardium, favoring slow conduction, promoting reentry circuits, and increasing overall susceptibility to ventricular arrhythmias. Differentiating adipose metaplasia from dense scarring is important for precise substrate-based ablation strategies. In this context, the integration of CT into EAM systems represents a valuable tool. CT allows for high-resolution differentiation between adipose tissue and fibrosis (Figure 4). When combined with CMR, which provides detailed tissue characterization LGE, CT enhances the accuracy of substrate identification. A multimodal approach leveraging CT and CMR integration into mapping systems can refine procedural planning and optimize ablation outcomes by distinguishing adipose metaplasia from scar tissue, ultimately improving guidance for mapping and ablation [45,46,80,81].

The remarkable potential of CMR to characterize scar tissue and to facilitate catheter ablation has been confirmed by various studies including an increasing number of patients in recent years.

PSI techniques can provide a comprehensive picture of the extent, transmurality, and heterogeneity of the underlying arrhythmic substrate by the use of automatic LGE analysis. CCs are often located in the border zone area of the scar with a complex tridimensional architecture [82]. Recently, the evolution of these techniques has provided additional information regarding the true arrhythmogenic potential of CCs in terms of their ramifications and transmurality [82]. Moreover, CMR-based analysis has been able to detect CCs demonstrated by double extra stimulus testing, even in areas with apparently normal EAM voltages [83].

In a prospective, experimental non-randomized study of 54 patients, color-coded PSI maps were obtained from a high-resolution 3Tesla LGE-CMR study and imported into the navigation system to aid VT substrate ablation [84]. The heterogeneous tissue channels (HTCs) depicted in the PSI were correlated to EAM information. In this study, the gold standard was the identification of channels through EAM, while HTCs in the scar were considered true positives if confirmed by mapping, showing 77% concordance. Channels identified by mapping but not by CMR were classified as false negatives (23%). There was also a 16% rate of false positives, where CMR identified an arrhythmogenic substrate not confirmed by mapping, and no ablation was performed in these cases. Compared to the control group, the integration of CMR with mapping resulted in reduced radiofrequency applications, increased non-inducibility rates, and improved arrhythmia-free survival during follow-up. Interestingly, false positives identified by CMR had more events at follow-up, suggesting that, in some cases, CMR might define the arrhythmogenic substrates even better than EAM.

Based on these intriguing results, the same group evaluated the feasibility and potential benefit of VT substrate ablation entirely guided by CMR imaging in a subsequent study [85], including patients with cardiac devices (excluded in previous studies). The authors prospectively compared a purely CMR-guided method using PSI maps alone with two historical approaches: standard EAM without CMR and EAM combined with CMR-derived PSI maps. The CMR-guided approach significantly shortened the procedure time, reduced fluoroscopy, and lowered the rate of inducible VT after ablation compared to the other methods. Over 12 months of follow-up, patients treated with CMR guidance showed fewer arrhythmia recurrences than those undergoing ablation with no CMR, with no differences between CMR-aided and -guided ablation. The study highlights the safety, efficiency, and improved outcomes of integrating advanced CMR imaging into VT substrate ablation, offering a promising direction for optimizing the management of scar-related arrhythmias.

**Figure 5:**
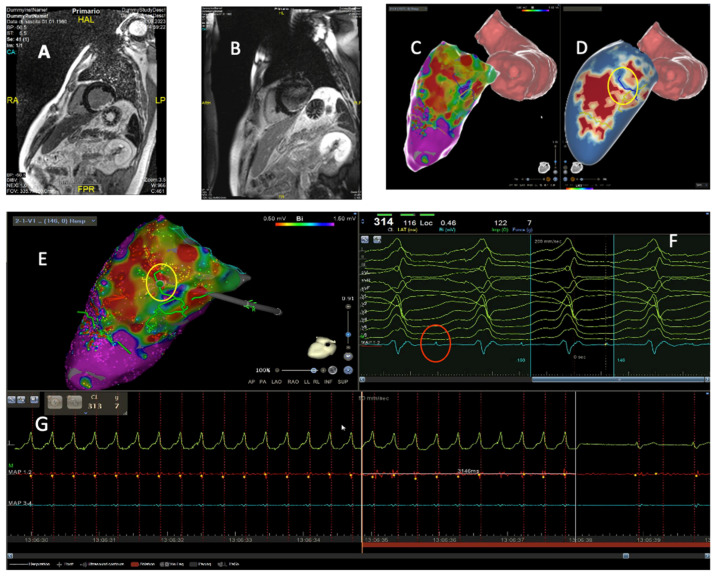
A patient with previous myocardial infarction experienced multiple ICD shocks due to recurrent episodes of VT. (**A**,**B**) The comparison of preprocedural CMR with (**A**) and without (**B**) WB sequences. The inferolateral scar was much more clearly delineated on the WB sequence than on the conventional one and was successfully imported into the EAM system using ADAS 3D software. (**C**) A posterior view of the bipolar voltage map with standard thresholds for dense scar (<0.5 mV) and border zone (<1.5 mV). (**D**) The corresponding projection of the color-coded LGE-CMR-derived PSI map (red = dense scar, blue = normal tissue, cream/orange = border zone). Putative conducting corridors were identified, annotated with yellow (10% layer) and blue (20% layer) lines, and superimposed onto the voltage map. (**E**,**F**) Following substrate ablation targeting LAVAs identified by EAM, VT was still inducible. The critical isthmus, exhibiting a diastolic potential ((**F**), red circle), was located in a region previously identified as an HTC on the PSI map ((**D**), yellow circle). (**G**) The application of radiofrequency energy resulted in prompt termination of the VT. ICD, implanted cardioverter defibrillator; VT, ventricular tachycardia; WB, wideband; CMR, cardiac magnetic resonance; EAM, electroanatomic mapping; LGE, late gadolinium enhancement; PSI, pixel signal intensity; LAVA, local abnormal ventricular activity; HTC, heterogeneous tissue channel.

**Figure 6:**
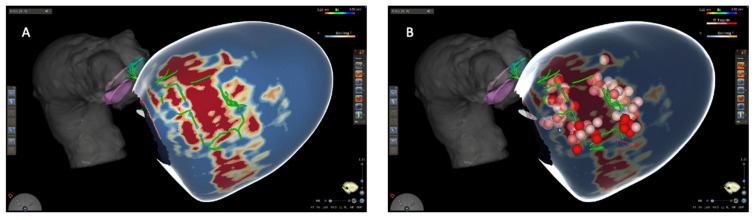
CMR-guided VT ablation in a patient with previous inferolateral MI and recurrent episodes of VT. (**A**) A color-coded LGE-CMR-derived PSI map. Blue: normal myocardium; red: dense scar; green lines: putative HTCs. (**B**) The dots indicate lesions set at the entrance and within the HCT. At the end of the procedure, VT was no longer inducible. After 36 months of follow-up, no arrhythmia recurrences occurred. CMR, cardiac magnetic resonance; MI, myocardial infarction; VT, ventricular tachycardia; LGE, late gadolinium enhancement; PSI, pixel signal intensity; HTC, heterogeneous tissue channel.

The VOYAGE trial [86] is an ongoing prospective, randomized, multi-center, open-label study with a control group, involving a total of eight centers. The primary objective is to analyze the outcome of CMR-guided and -aided approaches to VT ablation in terms of effectiveness at 12 months in comparison to a control group (standard-of-care VT ablation). The primary endpoint is defined as any VT recurrences during a 12-month follow-up. The enrollment has concluded and the results are expected in the next few months. Figure 5 and Figure 6 show two examples of CMR-aided and -guided VT ablation, respectively.

## 5. Future Perspectives

The CARTOSOUND FAM module is a new deep learning imaging algorithm integrated with ICE for the 3D reconstruction of cardiac anatomy without the need to manually annotate ultrasound (US) contours. This module is currently available for left atrium reconstruction [87,88]. If successfully applied to the ventricular chambers in the future, it could improve workflow efficiency and reduce operator dependency, as already highlighted in atrial fibrillation ablation.

The NuVision NAV is a 10 F ultrasound imaging catheter with a 4D ICE ultrasound transducer which conveys 3D location information that is integrated with the CARTO Navigation System (Biosense Webster), allowing for high-quality multiplanar reconstruction with minimal catheter manipulation. Four-dimensional ICE in a preclinical swine model demonstrated its ability to provide real-time multiplanar imaging and volumetric acquisition for guiding complex electrophysiology procedures. The technology allowed accurate electroanatomic reconstructions with minimal catheter movement and facilitated precise ablation of ventricular structures [89]. Potential benefits include reduced procedural time and improved safety.

Over the last few years, artificial intelligence (AI) applications in medical have dramatically accelerated, mainly due to the use of convolutional neural networks and transformer architecture. The possibility of extracting and analyzing huge amounts of data is particularly attractive for multimodality imaging and electrophysiological procedures, and it could significantly improve efficacy rates, cost, and the timing required [90,91]

These technological advancements are expected to further enhance the utility of multimodality imaging in VT ablation, providing electrophysiologists with more sophisticated tools for navigating complex cardiac structures.

## 6. Limitations

Despite the promising advancements in multimodal imaging for VT ablation, several limitations must be acknowledged. The use of ICE entails an additional procedural cost that may limit its widespread adoption. Image acquisition and processing can be time-consuming and technically demanding, requiring dedicated expertise and software integration, which may not be available in all centers. Moreover, while imaging modalities such as CT and PET offer high anatomical and metabolic detail, additional radiation exposure must be considered, especially in younger patients. The presence of implantable cardiac devices still represents a challenge for cardiac CMR despite the advent of WB sequences, which are not widely available. The heterogeneity of imaging protocols, lack of standardization across centers, and relatively small sample sizes in many studies reduce the generalizability of the current evidence.

## 7. Conclusions

Cardiac imaging has revolutionized the approach to VT ablation by offering precise anatomical and substrate characterization, improving procedural planning, and enhancing safety and efficacy. The integration of advanced imaging modalities with EAM enables a tailored, patient-specific strategy that may improve long-term outcomes, particularly in complex substrates. Although limitations persist, technological advancements such as the development of more automated image analysis tools, improved real-time integration capabilities, and deep-learning-based anatomical reconstructions promise to overcome current barriers. Future large prospective studies will be instrumental in defining the optimal role of imaging in VT ablation workflows and guiding the standardization of protocols. In the evolving field of electrophysiology, multimodal imaging is increasingly recognized as a key enabler of precision medicine.

## Data Availability

No new data were created or analyzed in this study. Data sharing is not applicable to this article.
